# WCEDSAM: A Lightweight Multi-Scale Colonoscopy Polyp-Segmentation Network Combining Frequency-Domain Decomposition and Adaptive Feature Enhancement

**DOI:** 10.3390/biology15090707

**Published:** 2026-04-30

**Authors:** Lei Wang, Tongyu Wang, Sitong Liu, Zheng Chen, Jie Zhang, Cong Jin, Dexing Kong

**Affiliations:** 1 School of Medical Technology and Information Engineering, Zhejiang Chinese Medical University, Hangzhou 310053, China; 13758582912@163.com (L.W.); wangtongyu0219@163.com (T.W.); jincong1122@outlook.com (C.J.); 2 School of Mathematical Sciences, Zhejiang Normal University, Jinhua 321004, China; 15990078352@163.com; 3 School of Basic Medical Sciences, Zhejiang Chinese Medical University, Hangzhou 310053, China; starar@126.com; 4 School of Pharmacy, Zhejiang Chinese Medical University, Hangzhou 310053, China; jiezhang0302@163.com; 5 School of Mathematical Sciences, Zhejiang University, Hangzhou 310058, China

**Keywords:** deep learning, polyp segmentation, bioinformatics, MedSAM, multi-scale

## Abstract

Colorectal cancer screening is challenged by variations in polyp morphology, indistinct polyp boundaries, and the high computational costs associated with current models. To address these issues, a lightweight medical image segmentation model, WCEDSAM, has been developed. WCEDSAM is based on a modified, compact version of MedSAM, which incorporates a Wavelet Transform-based component to extract and separate overlapping features at the pixel level. Additionally, a DSConv-ECA module is positioned before the ViT encoder to capture local features efficiently while reducing parameter count and enhancing inter-channel communication. Experimental results demonstrate that WCEDSAM achieves top performance on five public datasets, including Kvasir-SEG and CVC-ClinicDB, with 15.38 million parameters, achieving mean Dice (mDice) scores of 0.9383 on Kvasir-SEG and 0.9376 on CVC-ClinicDB. Cross-domain evaluations yield mDice scores of 0.9189 on CVC-ColonDB, 0.8961 on CVC-300, and 0.7765 on ETIS datasets, respectively, substantially outperforming other methods such as UNet++ and TransUNet.

## Simple Summary

The WCEDSAM model can accurately segment polyps in medical images. It is built on the MedSAM model and modified with two new components: WTCA, which integrates Wavelet Transform and Channel Attention to extract both high- and low-frequency information from an image and assign importance based on their respective contributions. The second component, DSConv-ECA, employs depthwise separable convolution and efficient-channel-aware attention to simplify the process while maintaining high performance. When operating simultaneously, these two modules not only handle multiple types of information but also improve the model’s robustness to minor variations, resulting in enhanced overall performance in practical applications. When evaluated on the public datasets Kvasir-SEG and CVC-ClinicDB, the model outperforms mainstream methods, including UNet++, Polyp_PVT, and PraNet, across both the Dice coefficient and precision-recall metrics. The operation is semi-automatic, eliminating the need for doctors to create rough masks manually, thereby replacing the previous manual annotation process.

## 1. Introduction

Colorectal cancer is the second leading cause of cancer-related mortality worldwide, and approximately 75% of cases originate from adenomatous polyps [1]. Traditional manual detection methods have proven inefficient, often resulting in missed lesions or inconsistent identification. Deep learning has emerged as a powerful tool for feature extraction and segmentation of complex medical images [2]; however, challenges remain due to the wide variability in polyp size, shape, and indistinct boundaries. Existing techniques face difficulties in integrating cross-layer features and constructing multi-scale feature representations, which complicates the adaptation of the model to varying feature scales. Consequently, small polyps may be overlooked, while larger polyps could be fragmented excessively during segmentation. This study aims to address these challenges by enhancing the model’s adaptive feature synthesis and improving inter-scale communication among multiscale components. This approach enhances contextual integration, thereby improving polyp segmentation performance. This improvement facilitates the detection of small polyps and contributes to the reduction of colorectal cancer risk [3].

Medical image segmentation is a challenging task in the field of medical imaging and image processing. Its objective is to delineate regions of interest in medical images corresponding to anatomical structures or pathological areas, thereby providing a foundation for clinical diagnosis and biomedical research. This enables more accurate diagnoses and supports optimal clinical decision-making [4,5]. Numerous methods have been developed for medical image analysis, including Convolutional Neural Networks (CNNs), Transformers [6], and other deep learning approaches. CNNs, a class of deep feedforward neural networks, utilize convolutional operations for feature extraction. Filters or kernels are applied to the input image, sliding across its spatial dimensions to compute feature maps, thereby enabling hierarchical representation learning. This approach effectively reduces the dimensionality of large-scale image recognition problems, rendering them computationally tractable for training. However, CNNs can be challenging to implement and require extensive training time. Consequently, the Transformer architecture, which relies on an attention mechanism, was developed. Transformers are designed to replace traditional sequential models, such as RNNs [7] and LSTMs [8], enabling the effective modeling of long-range dependencies.

Transformer models offer the advantage of enabling efficient parallel computation. However, they require substantial CPU cycles and memory, which can pose challenges on devices or environments with limited computational resources. Furthermore, architectures based on the self-attention mechanism may experience reduced effectiveness for long sequences, as the computation of attention weights scales quadratically with sequence length. In response, several enhanced models, including TransUNet [9], TransFuse [10], and Polyp_PVT [11], have been developed to improve medical imaging segmentation. The combination of Transformer and CNN strengths facilitates enhanced local feature extraction while preserving global contextual information, thereby achieving an improved trade-off between segmentation accuracy and computational efficiency. For imaging segmentation tasks, SAM [12] performs well on natural images but exhibits limitations when applied to medical images. MedSAM [13] adapts SAM for application in medical imaging and has now emerged as a versatile tool for segmenting diverse types of medical images.

The challenges in medical imaging segmentation include several critical issues. Objects in images may vary significantly in size, complicating their accurate segmentation [14,15,16]. Feature information from adjacent small regions can be highly similar, making boundary delineation difficult [17,18,19]. Consequently, defining precise boundaries between adjacent structures is challenging. Furthermore, the high similarity of features among neighboring patches further increases segmentation difficulty. Additionally, intra-image non-uniformity in object representation may lead to inconsistent segmentation results. Specifically, in small-sized images, often generated by downsampling, edge and fine structural information may be lost, resulting in blurred segmentation boundaries or missing object regions [20,21,22]. Small lesions, particularly those under 5 mm, are difficult to distinguish from surrounding tissue and may be entirely missed during segmentation [23]. Lesions of the same type may exhibit diverse characteristics across scales, and size variations are challenging to capture in lower-resolution images. Thus, accurate segmentation of small-scale images remains a significant challenge.

A novel lightweight model, WCEDSAM, is proposed. This model is designed to address challenges associated with multiscale medical images. The WCEDSAM module integrates a Wavelet Transform with a channel attention mechanism. Wavelet decomposition is applied to the registered input image, using pass-band filters to separate high- and low-frequency components. These components are individually processed to enhance feature representation [24,25]. Features are extracted within their respective frequency domains and subsequently merged. Finally, the two-dimensional discrete Wavelet Transform (DWT) is employed to reconstruct the image from the transformed coefficients. This process yields the final merged image. Simultaneously, a channel attention strategy is applied, allowing the model to adaptively weight each channel according to its feature significance. This enhances the feature maps, improving the model’s sensitivity in regions of interest. The combination of Wavelet Transform and Channel Attention enables the extraction of detailed features while reducing noise.

It is noteworthy that several exploratory studies have investigated the integration of wavelet transforms and attention mechanisms for medical image segmentation. WBANet [26] introduces a Wavelet-Guided Feature Enhancement (WFE) module, in which low- and high-frequency information obtained from the wavelet transform is processed through separate convolutional layers. Learnable coefficients are employed to dynamically guide feature optimization, thereby enabling synergistic enhancement of both global structures and edge details in polyp segmentation. CFWANet [27] proposes a cross-scale fusion and wavelet aggregation network featuring a two-branch architecture for separate processing of multi-scale features. Wavelet transforms are utilized to capture multi-resolution information, thereby enhancing boundary detection capabilities. Furthermore, WRANet [28] integrates the discrete wavelet transform into the downsampling process. By separating low- and high-frequency components and incorporating an attention mechanism, it reduces feature loss and demonstrates superior performance in both aneurysm and polyp segmentation. WaveFormer [29] additionally directs self-attention computations to the low-frequency subbands produced by wavelet decomposition, substantially reducing computational overhead while retaining high-frequency details necessary for precise boundary delineation. In contrast, the WTCA module proposed in this study innovatively constructs a cross-subband channel-cooperative attention mechanism. By explicitly modeling channel dependencies between different frequency subbands, it achieves deep interaction and adaptive fusion of frequency-domain features, thereby enabling the efficient capture of high-frequency details even under low-resolution input conditions.

ECANet [30], an efficient channel attention module designed for CNNs, is presented. A small one-dimensional convolutional (1D Conv) layer is introduced to enable the network to selectively emphasize channel-specific information without significantly increasing computational cost. Although traditional global average pooling captures the overall feature information of each channel, it fails to account for inter-channel relationships. By combining global average pooling with an additional one-dimensional convolutional layer, ECANet can model the dependencies between channels. This mechanism enables ECANet to more effectively assess the relative importance of channel features. Depthwise-separable convolutions [31] consist of two operations: depth-wise and point-wise convolutions. Spatial features are extracted via depth-wise convolutions, while channel features are captured using point-wise convolutions. Depthwise-separable convolutions perform convolutions within channel groups along the feature dimension. Separate depth-wise convolutions are applied to each channel, followed by a 1 × 1 point-wise convolution to aggregate the channels before output. By integrating ECANet with depthwise separable convolutions, WCEDSAM reduces computational cost while effectively representing features through the extracted convolutional components.

**Figure 1:**
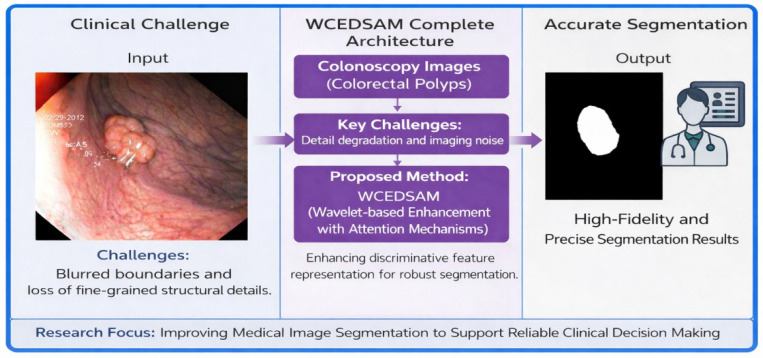
We have developed a novel deep learning segmentation network, WCEDSAM, for image segmentation to assist clinicians in lesion diagnosis.

When combined, these two modules provide complementary advantages, enabling the network to capture comprehensive information while maintaining computational efficiency. This approach, combining the two-module channels with ECANet, assigns adaptive weights to enhance the detection of regions of interest, even in complex scenarios. The proposed WCEDSAM utilizes an initial rough mask to facilitate the segmentation task, assisting clinicians in lesion diagnosis from medical images. Clinically, this represents a significant advancement for practical hospital applications, as illustrated in the corresponding diagram, as shown in Figure 1.

## 2. Related Works

### 2.1. CNN in Medical Imaging Segmentation

Medical imaging serves as a quantitative diagnostic support tool. Medical image segmentation encounters numerous challenges, including variations in object shape, blurred boundaries, multi-scale structures, and limited availability of annotated data. In recent years, deep learning, particularly models based on the encoder–decoder architecture, has achieved significant advancements.

Attention U-Net [32] proposed by Ozan Oktay et al. automatically identifies structures of varying shapes and sizes, highlighting regions relevant for specific tasks. UNet++ [33] proposed by Zhou et al. incorporates densely nested skip connections between the encoder and decoder subnetworks to facilitate feature map integration. Compared to UNet [34], UNet++ has a more complex architecture, a larger number of parameters, and a slower training speed. Ange Lou et al.’s proposed CaraNet [35] is designed for the segmentation of small-scale objects in medical images. It employs a partial decoder to localize polyps, uses the Contextual Feature Pyramid (CFP) to adapt to objects of varying sizes, and enhances polyp segmentation capabilities through the A-RA module. However, CaraNet exhibits limitations in capturing fine details and performs optimally only with large amounts of high-quality annotated images. In regions with sparse information or inconsistent annotations, segmentation performance may be compromised. By incorporating a reverse attention module, PraNet accurately captures boundary features from the initial segmentation map [36]. ACSNet adaptively fuses global and local contextual information to improve polyp segmentation performance [37]. SANet employs a shallow attention module and color augmentation to extract shallow features, ensuring robust segmentation results [38]. UACANet [39], proposed by Taehun Kim et al., captures boundary information in uncertain regions using a contextual attention module, enhancing polyp segmentation accuracy. ConDSeg, developed by Lei et al. [40], addresses soft boundaries and collinear structures commonly encountered in medical images. A consistency reinforcement pretraining strategy enhances the encoder’s robustness against external perturbations, including unclear boundaries and poor contrast. EMCAD [41], put forth by Md Mostafijur Rahman et al., employs multi-scale deep convolutional blocks to extract features at multiple scales, facilitating accurate medical image segmentation with improved efficiency. Additionally, lightweight architectural designs have been investigated to enable efficient deployment on resource-constrained devices [42].

Although some deep learning approaches have been applied for target segmentation in medical imaging, no existing model can perfectly detect and segment small lesions, blurred boundaries, or fine structures. Furthermore, these methods are highly dependent on the quality and quantity of annotated data. In medical imaging, data scarcity, high annotation costs, and inconsistencies in labeling pose additional challenges. Moreover, effectively capturing multi-level features and efficiently integrating deep and shallow representations remains an unresolved challenge in medical imaging analysis.

### 2.2. Transformer in Medical Imaging Segmentation

The attention mechanism remains a central component of transformer architectures for natural language processing. By computing correlations between elements of an input sequence, attention allows each element to access information from all other elements, thereby enabling effective modeling of long-range dependencies. In the basic form, the input sequence is linearly projected into queries, keys, and values. The similarity between each query and all keys is computed, normalized to obtain attention weights, and used to perform a weighted aggregation of the value vectors, producing the output. To prevent the model from accessing future positions during training and to capture multiple types of relationships simultaneously, masking and multi-head attention are employed. Specifically, multi-head attention allows the model to jointly attend to information from different representation subspaces.

(1) $$\mathrm{MultiHead}(Q,K,V)=\mathrm{Concat}({\mathrm{head}}_{1},{\mathrm{head}}_{2},\ldots ,{\mathrm{head}}_{h})W^{O}$$

Each attention head performs its own self-attention computation, allowing the model to capture global information across different regions. Because self-attention excels at global modeling, it was rapidly incorporated into the field of computer vision. Dosovitskiy et al. demonstrate that Vision Transformers achieve high performance on numerous image-classification tasks by partitioning images into equally sized patches and applying standard Transformer encoding to extract relevant features. The success of ViT indicates that models based solely on attention mechanisms can learn long-range dependencies within images, even in the absence of traditional convolutional operations. This represents a novel architectural design paradigm for a visual task.

TransUNet, proposed by Chen Jieneng et al., enhances medical image segmentation performance through the integration of a Transformer model. Transformers achieved significant success in NLP, primarily due to their ability to model long-range dependencies. The introduction of self-attention mechanisms and global contextual information improves both the accuracy and speed of medical image segmentation. TransFuse, proposed by Zhang Yundong et al., simultaneously employs a shallow CNN encoder and a Transformer-based segmentation network. It utilizes a BiFusion module to simultaneously fuse features from both branches for joint prediction. The model captures both low- and high-level spatial structures as well as semantic information. This approach eliminates the need for deep neural networks, mitigating issues such as vanishing gradients and feature underrepresentation, while also reducing model size and inference time.

The Polyp-PVT method, proposed by Dong Bo et al., extracts both high-level and low-level features via the cascade fusion, camouflage recognition, and similarity aggregation modules, which are then fused to produce the final output. These modules facilitate the capture of polyp details, including textures, colors, and structural features. Global attention mechanisms are employed to embed these detailed visual features into higher-level semantic representations.

Medical image segmentation and self-attention mechanisms, along with their variations, have been commonly employed to enhance feature representations of objects with varying shapes, blurry boundaries, and multiple scales. For example, CFANet [43] incorporates a channel-feature attention module that combines both channel and spatial attention and utilizes dual-phase features for adaptive context fusion, thereby improving segmentation accuracy. In contrast, MADGNet [44] integrates multi-scale attention and a gating mechanism to adaptively fuse multi-scale features while mitigating noise interference. SAM3-UNet [45] achieves efficient fine-tuning of SAM3 [46] through the incorporation of a lightweight adapter positioned between the frozen SAM3 image encoder and a UNet-style decoder. Studies have demonstrated that combining convolutional neural network-based feature extraction with attention mechanisms produces significant improvements in the field of medical imaging [47]. The aforementioned methods also address issues associated with coarse or failed predictions of fine structures and contextual objects while maintaining low computational complexity. However, transformer-based methods require extensive training data and high computational resources, making their application challenging in medical imaging scenarios where data are scarce.

## 3. Materials and Methods

In this work, a novel medical image segmentation model, WCEDSAM, is introduced as a lightweight and performance-enhanced version of the existing baseline model, MedSAM. Although the MedSAM model exhibits exceptional segmentation capabilities, its high computational cost and large memory requirements pose challenges when processing high-resolution medical images. To address common clinical constraints, such as limited GPU memory, the standardized input image size was set to 352 × 352 pixels. This input size reduces computational burden while providing sufficient information for effective processing.

**Figure 2:**
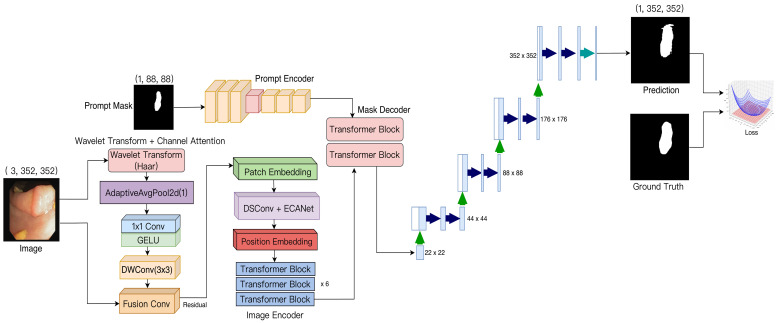
We present a Transformer-based medical image segmentation model architecture that first extracts multiscale image features using Haar wavelet transforms and channel attention mechanisms. After patch embedding, these features are fed into an image encoder comprising DSConv and ECANet. At the same time, the Prompt Mask, after being processed by the Prompt Encoder, is fed into the Mask Decoder, which contains six Transformer blocks along with the output from the image encoder, ultimately generating a segmentation prediction result against which the loss is calculated relative to the ground truth.

The overall structure of WCEDSAM is illustrated in Figure 2. It primarily comprises five components: (1) a pre-processing module employing wavelet transform and channel attention (WTCA), (2) a DSConv_ECA block, which integrates depthwise separable convolutions with ECA-Net attention fusion, (3) an image encoder, (4) a prompt encoder, and (5) a mask decoder. The WTCA module is positioned at the initial stage of the network to process the input image and extract multi-scale frequency-domain feature information. The DSConv_ECA block functions as a subordinate module preceding the vision transformer encoder, serving as a low-level feature extractor. Simultaneously, the prompt encoder and mask decoder adhere to the original MedSAM design, being responsible for processing interactive prompts and generating the final segmentation outputs, respectively. The mathematical representation of this architecture can therefore be formally expressed as follows:

(2) $$F_{WCEDSAM}(I,M_{p})=D(E_{i}(B(W(I))),E_{p}(M_{p}))$$

Here, $I\in R^{3\times 352\times 352}$ represents the input image, $M_{p}\in R^{1\times 88\times 88}$ represents the prompt mask, W denotes the WTCA module, B denotes the DSConv-ECA module, $E_{i}$ denotes the image encoder, $E_{p}$ denotes the prompt encoder, and D denotes the mask decoder.

### 3.1. Wavelet Transform and Channel Collaborative Attention Module (WTCA)

**Figure 3:**
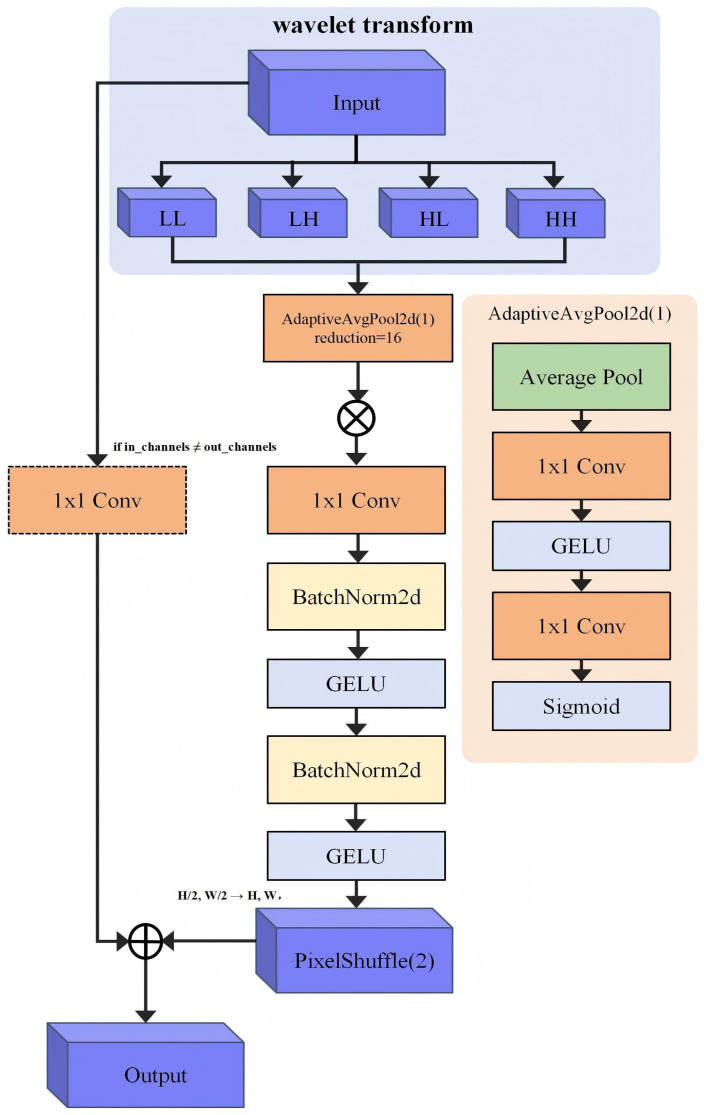
We present an image processing framework that combines the wavelet transform with the Channel Attention mechanism. The input image is decomposed into four subbands—LL, LH, HL, and HH—via a wavelet transform. Their features undergo channel weight optimization through a Channel Attention module comprising average pooling, 1 × 1 convolution, ReLU, and Sigmoid. Following this, the features undergo 1 × 1 convolution, batch normalization, ReLU, and bilinear upsampling, and are then fused with the residual from the original input to produce the final output.

The wavelet decomposition transforms signals into subbands of varying frequencies, making it highly applicable in medical imaging. Lesional regions in medical images typically exhibit higher variance within specific frequency subbands. For example, textural changes in tumors are more pronounced in high-frequency subbands, whereas low-frequency subbands preserve the overall organ shape. Based on this observation, the WTCA module was developed and integrated at the beginning of the network to enhance feature representation in the frequency domain. The architecture of the WTCA module is illustrated in Figure 3.

#### 3.1.1. Two-Channel Wavelet Decomposition

Given an input feature map $X\in R^{B\times C\times H\times W}$, a two-dimensional discrete-wavelet transform (DWT) is first applied to each channel using the Haar wavelet basis, resulting in the decomposition of the image into four subbands:

(3) $$\left\{LL^{\left(b,c\right)},LH^{\left(b,c\right)},HL^{\left(b,c\right)},HH^{\left(b,c\right)}\}=DWT2(X^{\left(b,c\right)}\right)$$

Here, $\mathrm{LL}\in R^{H/2\times W/2}$ represents the low-frequency approximation component, while $\mathrm{LH},\mathrm{HL},\mathrm{HH}\in R^{H/2\times W/2}$ represent the high-frequency detail components along the horizontal, vertical, and diagonal directions, respectively. After applying this operation to all samples and channels in the batch, the reconstructed tensor $C\in R^{B\times 4C\times H/2\times W/2}$ is obtained.

#### 3.1.2. Channel-Cooperative Attention Mechanism

Global average pooling of raw spatial features, as employed by traditional attention mechanisms, fails to capture differences in the frequency domain. Accordingly, a cross-subband channel-cooperative attention mechanism was designed as follows. First, a global context aggregation operation is performed on the multi-band features, which are concatenated to dimensions H′ = H/2 and W′ = W/2:

(4) $$Z=\frac{1}{H^{\prime }W^{\prime }}\sum_{i=1}^{H^{\prime }}\;\;\;\;\sum_{j=1}^{W^{\prime }}C_{:,:,i,j}$$

Two additional processing paths were employed on these features. In the first path, the features undergo dimensionality reduction through 1 × 1 convolutions, followed by a GELU activation and normalization via BatchNorm2d. Subsequently, an additional 1 × 1 convolution and a Sigmoid activation function are applied to generate the channel attention weights. In the second branch, the features are processed through a 1 × 1 convolution, followed by a GELU activation and BatchNorm normalization. Finally, a GELU activation is applied once more, with the resulting output fed into the subsequent layer for feature fusion.

Specifically, if discrepancies exist between the input and output dimensions, an additional 1 × 1 convolution is applied. The dimensionality reduction ratio in the attention module is set to r = 16. Inter-channel dependencies are modeled using two layers of 1 × 1 convolutions. The resulting attention weights $A\in R^{B\times 4C\times 1\times 1}$ are employed to recalibrate the relative importance of each frequency band:

(5) $$C^{\prime }=C⊗A$$

Finally, the weighted features are processed through 1 × 1 convolutions, added to the output of the original input, and connected via a residual connection to produce the final output:

(6) $$Y=F_{\mathrm{fusion}}(C^{\prime })+F_{\mathrm{residual}}(X)$$

For the remaining components, two successive pixel shuffle operations are applied to restore the spatial dimensions from (H/2, W/2) to the original input size (H, W). This residual architecture ensures effective gradient propagation, even when certain augmentation operations are bypassed within the network.

### 3.2. Deep Separable Convolution with ECA Module (DSConv-ECA)

Prior to the Vision Transformer encoder, a lightweight feature preprocessing module was introduced, integrating the efficiency of depthwise separable convolutions with the channel modeling capabilities of ECA. This design is motivated by the observation that patch embedding operations in Transformer encoders can result in the loss of local details, whereas medical image segmentation tasks are highly sensitive to edges and fine structural information. As illustrated in the figure, the DSConv-ECA module is designed to preserve local structural information while simultaneously enhancing the expressive capacity of channel features.

#### 3.2.1. Depth-Separable Convolutions

Depthwise separable convolution is employed to decompose standard convolution into two distinct operations: depthwise convolution and pointwise convolution. This approach significantly reduces the number of parameters while preserving the capacity for feature extraction. For an input feature $X\in R^{C\times H\times W}$, spatial filtering is performed independently on each input channel by a deep convolutional network.

(7) $$Y_{dw}={DepthwiseConv2D}_{k=3}(X)$$

Here, k = 3 denotes the size of the convolutional kernel. Subsequently, pointwise convolution is applied using a 1 × 1 convolution to integrate information across channels:

(8) $$Y_{pw}={Conv2D}_{1\times 1}(Y_{dw})$$

Compared to standard convolutions, the number of parameters in depthwise separable convolutions is expressed as: $P_{ds}=C\cdot k^{2}+C\cdot C^{\prime }\cdot 1^{2}$. When the number of output channels C′ is large, the parameter count is approximately $\frac{1}{C^{\prime }}+\frac{1}{k^{2}}$ times that of a standard convolution, resulting in a significant reduction in computational overhead.

#### 3.2.2. Efficient Channel Attention (ECA)

The ECA module captures local cross-channel interactions without reducing dimensionality, thereby avoiding the computational cost associated with the fully connected layers in SENet. Its core principle is to enable information flow across channels through one-dimensional convolutions, as illustrated in the ECA block below. First, global average pooling is applied to the feature map to obtain the channel-wise statistics:

(9) z=AdaptiveAvgPool2D(X)

Subsequently, the channel attention weights are obtained via a one-dimensional convolution with a kernel size k, where the kernel size k is adaptively determined as follows:

(10) $$k=\psi (C_{\mathrm{out}})={\left|\frac{{log}_{2}(C_{\mathrm{out}})+b}{\gamma }\right|}_{\mathrm{odd}}$$

In the experiment, the parameters were set as γ = 2 and b = 1, ensuring that k remained an odd number. The attention weights were then computed as follows:

(11) $$a=\sigma ({Conv1D}_{k}(z))$$

The final output is obtained as the channel-wise weighted sum of the original features and the attention weights, expressed as: O = X × s. Additionally, the module incorporates normalization structures analogous to LayerNorm, including channel-wise mean computation, subtraction, and squaring operations, to improve training stability.

#### 3.2.3. Module Structure and Residual Connections

**Figure 4:**
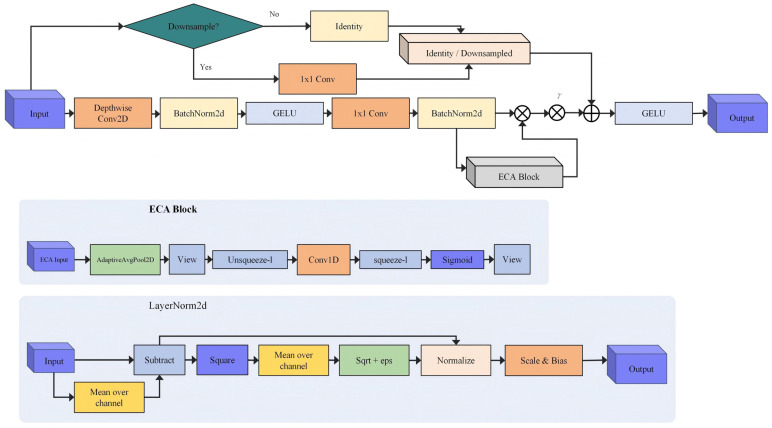
We present a neural network module architecture that integrates depthwise separable convolutions and attention mechanisms. The module initially processes input features through depthwise convolutions (Depthwise Conv2D), batch normalization (BatchNorm2D), and the GELU activation function, followed by channel dimension adjustment via 1 × 1 convolutions. At the same time, the input features are passed through the conditional sampling branch (which contains a 1 × 1 convolution or an identity mapping) and then added to the features from the main branch, forming a residual connection. The ECA block embedded within the module generates channel attention weights via adaptive average pooling (AdaptiveAvgPool2D) and 1D convolutions and applies channel-weighted averaging to the feature maps. In addition, the figure provides a detailed illustration of the computational process for LayerNorm2D, including subtraction of the mean, squaring, variance calculation, and normalization operations for each channel dimension.

The overall architecture of the DSConv_ECA module is illustrated in Figure 4. The input is initially processed through deep convolution and pixel-wise convolution, followed by BatchNorm2d and the GELU activation function. Subsequently, the data passes through a 1 × 1 convolution and BatchNorm2d prior to entering the ECA block, which performs channel attention modeling. The module as a whole utilizes a residual connection mechanism, as described below:

(12) Output=Input+ECA(Conv(Input))

The module increases the number of input channels from 3 to 64, thereby supplying the subsequent Transformer encoder with a comprehensive set of low-level visual features. Owing to the lightweight design of deep separable convolutions combined with ECA, the DSConv_ECA module requires approximately one-ninth of the parameters of conventional convolutions, achieving both high computational efficiency and robust feature representation.

### 3.3. Encoders and Decoders

#### 3.3.1. Image Encoder

The image encoder employs a Vision Transformer architecture and is tasked with extracting high-level semantic features from the enhanced input. Let X denote an input feature map of dimensions 3 × 352 × 352. Initially, it is partitioned into 22 × 22 visual tokens using 16 × 16 patch embeddings, after which spatial information is incorporated through learnable position encodings:

(13) $$Z_{0}=\left[x_{class};x_{1}W_{p};x_{2}W_{p};;x_{N}W_{p}\right]+E_{pos}$$

Here, $W_{p}\in R^{3\times 384}$ denotes the projection matrix, and $E_{pos}\in R^{\left(N+1\right)\times 384}$ represents the position encoding, where N = 484. The features are subsequently processed through six Transformer blocks, each comprising six self-attention heads and a multi-layer perceptron (MLP) feedforward network:

(14) $$Z\prime_{l}=MHA\left(LN\left(Z_{l-1}\right)\right)+Z_{l-1}$$

(15) $$Z_{l}=MLP\left(LN\left(Z\prime_{l}\right)\right)+Z\prime_{l}$$

Following the patch embedding stage, the DSConv_ECA module is incorporated to enlarge the receptive field and enhance feature representation through the use of deep separable convolutions combined with an efficient channel attention mechanism. The resulting output feature, $Z_{l}\in R^{384\times 22\times 22}$, is subsequently supplied to the decoder as an image embedding.

#### 3.3.2. Prompt Encoder

The prompt encoder is responsible for converting user interactions, such as click selections and masking, into corresponding embedding vectors, which are aligned in parallel with the image features. In this semi-automatic segmentation framework, input prompts are employed to generate an initial coarse segmentation through morphological erosion. This approach substantially reduces the size of the original mask provided by users and enables the model to more accurately delineate the region corresponding to the desired boundary, rather than relying solely on a few annotated points, thereby allowing precise segmentations even when minimal human corrections are necessary. The hint mask is subsequently post-processed using a three-layer downsampling network to rescale it to the appropriate resolution corresponding to the image features:

(16) $$E_{p}=f_{\mathrm{down}}(M_{p})\in R^{384\times 22\times 22}$$

#### 3.3.3. Mask Decoder

The mask decoder employs a lightweight Transformer that integrates image features with prompt information and progressively upsamples the result to the original resolution. This procedure can be divided into three stages: Initially, the image feature $E_{i}\in R^{384\times 22\times 22}$ is combined element-wise with the prompt feature E_p_, and a two-dimensional sinusoidal position encoding $P\in R^{384\times 22\times 22}$ is incorporated, yielding $F_{0}=E_{i}+E_{p}+P$. The position encoding is computed according to the standard sine and cosine formulations:

(17) $$P_{\left(2i,y,x\right)}=sin\left(\frac{y}{{10,000}^{2i/384}}\right)$$

(18) $$P_{\left(2i+1,y,x\right)}=cos\left(\frac{y}{{10,000}^{2i/384}}\right)$$

An identical formulation is applied along the horizontal dimension. The resulting features are subsequently flattened and integrated using two Transformer blocks:

(19) $$F_{1}=TransformerBlock\left(LayerNorm\left(F_{0}\right)\right)$$

(20) $$F_{2}=TransformerBlock\left(LayerNorm\left(F_{1}\right)\right)$$

Each Transformer block comprises a self-attention mechanism and a feedforward network with a multi-layer perceptron (MLP) expansion ratio of 4. Finally, the resolution is progressively restored using a fivefold upsampling module. Each upsampling stage incorporates a transposed convolution, layer normalization, and a GELU activation function. Ultimately, a 1 × 1 convolution generates a single-channel segmentation mask $\hat{M}\in R^{1\times 352\times 352}$. This progressive upsampling strategy mitigates the checkerboard artifact while progressively reconstructing fine-grained details.

### 3.4. Training and Optimization

WCEDSAM is trained using an end-to-end approach, with a composite loss function that integrates Dice loss and binary cross-entropy loss:

(21) $$L=\lambda_{d}L_{dice}+\lambda_{b}L_{bce}$$

In this study, the weighting factors are set as λ_d_ = 1.0 and λ_b_ = 1.0. The Dice loss is employed to encourage regional consistency:

(22) Ldice=1−2∑iM^iMi+ϵ∑iM^i+∑iMi+ϵ

The binary cross-entropy loss is employed to provide pixel-wise supervision:

(23) $$L_{bce}=-\frac{1}{N}\sum_{i}\left[M_{i}log\left({\hat{M}}_{i}\right)+\left(1-M_{i}\right)log\left(1-{\hat{M}}_{i}\right)\right]$$

The model is trained using the Adam optimizer with an initial learning rate of 1 × 10^−4^, a weight decay of 1 × 10^−5^, and a cosine annealing learning rate schedule. All experiments were conducted using PyTorch 2.4.0, employing a batch size of 8 and 100 training epochs. Gradient norms were clipped to a maximum value of 1.0 to ensure training stability. This architecture reduces computational requirements while maintaining effective segmentation performance when combined with the MedSAM method. Consequently, the model can be deployed on devices with limited memory by directly processing inputs at a resolution of 352 × 352. This facilitates potential clinical applications, demonstrating the practical utility of the model.

## 4. Results

### 4.1. Dataset

The public open-source Kvasir-SEG dataset [48], which contains 1000 endoscopic images of colorectal polyps and their corresponding precise masks, was utilized. To evaluate model performance across different imaging modalities and lesions in various anatomical regions, a publicly available dataset was introduced. Following standard machine learning procedures, the dataset was partitioned into training and testing sets at an 8:2 ratio. Public datasets that facilitate comparison with prior research and provide diverse testing cases are preferred to enable a more robust evaluation of the model’s generalizability.

The freely accessible CVC-ClinicDB dataset [49], comprising 612 high-resolution images obtained from 29 colonoscopies, was utilized. Each image is accompanied by highly accurate, pixel-level ground truth annotations delineating all polyp regions. In this study, the dataset was partitioned into training and testing sets at an 8:2 ratio, resulting in 489 images allocated for training and 123 images reserved for independent testing.

The CVC-ColonDB dataset [50] was utilized to evaluate the cross-dataset generalization capability of polyp segmentation models. This dataset comprises 380 high-quality colonoscopy images obtained from 15 separate clips, each with a resolution of 574 × 500 pixels. In CVC-ColonDB, all 380 images are reserved exclusively for testing, with no overlap with the training data, thereby providing a rigorous assessment of model performance on out-of-distribution instances.

The CVC-300 dataset [51] was utilized to evaluate our model’s performance in detecting polyps in colonoscopic images. It comprises 60 static images selected from 44 colonoscopy sequences, each with a resolution of 574 × 500 pixels. As a cross-domain generalization testing set, it has been used exclusively for evaluation in prior studies and was not employed for training.

The ETIS dataset [52] was used exclusively during the testing phase to evaluate the model’s domain adaptability. It contains 196 high-quality colonoscopy images in the testing set, each with a resolution of 1225 × 966 pixels, comprising a total of 208 polyps. Performance variations observed in this dataset when evaluated under different scenarios serve as an important indicator of the clinical applicability of polyp segmentation methods. Information on the five datasets is shown in Table 1.

### 4.2. Evaluation Metrics

In this study, the performance of WCEDSAM on ultrasound images was evaluated by categorizing the data into multiple groups. Four specific metrics were employed to assess the models: the Dice Similarity Coefficient (DSC), Intersection over Union (IoU), precision, and recall. These metrics provide a comprehensive evaluation of the predictions from multiple perspectives, including overall accuracy, edge alignment, and class-specific recognition, thereby offering a more complete assessment of model performance.

#### 4.2.1. Dice Coefficient

The Dice coefficient is a widely used performance evaluation metric in medical image segmentation, which quantifies the overlap between predicted and ground-truth regions. Mathematically, it is defined as twice the size of the intersection between the predicted region and the ground truth, divided by the sum of their areas. This metric is sensitive to the extent of overlap within the interior regions and is therefore well-suited for assessing the accuracy of target segmentation. The Dice coefficient is calculated using the following formula:

(24) $$Dice=\frac{2\times |P\cap G|}{\left|P|+|G\right|}$$

Here, p represents the segmentation result obtained from the model’s binary output, g denotes the ground truth mask, providing the accurately labeled reference, and |.| indicates the number of elements or pixels. The Dice coefficient ranges from 0 to 1, with higher values indicating a better agreement between the predicted segmentation and the ground truth.

#### 4.2.2. Intersection over Union

The intersection-over-union (IoU) ratio, commonly referred to as the Jaccard index, is employed to quantify the proportion of the predicted region that overlaps with the ground truth region. Although this metric evaluates the area of overlapping regions, it is highly sensitive to minor discrepancies along segmentation boundaries compared to the Dice coefficient. The corresponding formula is provided below:

(25) $$IoU=\frac{\left|P\cap G\right|}{\left|P\cup G\right|}$$

A monotonic relationship exists between the intersection over union (IoU) and the Dice coefficient, expressed by the formula $\mathrm{Dice}=\frac{2\times \mathrm{IoU}}{1+\mathrm{IoU}}$. Nevertheless, the two metrics are not numerically identical, as the IoU value is generally slightly lower than the corresponding Dice score.

#### 4.2.3. Precision

Accuracy, or the positive predictive value, quantifies the proportion of pixels predicted as lesions that actually correspond to polyps. This metric serves to evaluate the predictive quality of the model. True positives (TP) denote correctly predicted foreground pixels, while true negatives (TN) represent correctly identified background pixels. False positives (FP) correspond to background pixels incorrectly classified as foreground, and false negatives (FN) indicate foreground pixels mistakenly labeled as background. A higher accuracy implies that the model produces minimal noise and a limited number of misclassified foreground pixels. The formula is defined as follows:

(26) $$Precision=\frac{TP}{TP+FP}$$

#### 4.2.4. Recall

Recall, also referred to as sensitivity or true positive rate, is defined as the ratio of correctly predicted positive observations to the total number of actual positive instances. The detection performance evaluated using recall indicates the proportion of the actual polyp area that has been correctly identified by the algorithm. A high recall value suggests that few polyp regions were missed, indicating that the polyps in the image are well captured by the model. It reflects the model’s ability to correctly identify all relevant regions of interest. Recall is calculated as follows:

(27) $$Recall=\frac{TP}{TP+FN}$$

### 4.3. Data Augmentation

To reduce overfitting commonly observed in deep learning models during medical image segmentation and to enhance the model’s generalization to variations in lesion morphology and location, targeted data augmentation strategies are introduced during the training phase. Specifically, a probability-triggered random spatial transformation mechanism is designed, which applies synchronized geometric transformations to both the input image and its corresponding ground-truth mask, thereby preserving spatial consistency.

Regarding specific augmentation operations, this strategy primarily employs two types of geometric transformations. First, random horizontal and vertical flipping is applied, each with a trigger probability of 0.5, to mitigate directional uncertainty (up-down or left-right) in endoscopic images arising from variations in camera angle. Second, a random rotation operation is applied with a trigger probability of 0.3, and the rotation angle is sampled randomly within the range of −15° to 15°. This small-angle rotational perturbation effectively simulates perspective shifts induced by minor endoscope tremors during clinical procedures, thereby improving the model’s robustness to local deformations.

Regarding specific augmentation operations, this strategy primarily employs two types of geometric transformations. It should be noted that the described data augmentation operations are applied exclusively during the training phase and are disabled during testing to maintain the objectivity of evaluation results. Furthermore, all geometric transformations are applied simultaneously to both the input image and its corresponding mask, thereby ensuring precise pixel-level alignment of the labels. This multidimensional, low-probability perturbation-based data augmentation strategy not only effectively increases the diversity of training samples without additional annotation costs but also enables the model to learn more discriminative rotation- and flip-invariant features, thereby establishing a foundation for improved segmentation accuracy in complex scenarios.

### 4.4. Comparative Experiment

The proposed architecture was implemented using the PyTorch deep learning framework. The software environment included PyTorch version 1.10.0, Python 3.8.12, and CUDA 10.2. Training was performed on an NVIDIA GeForce GTX 1080 Ti GPU.

In this study, a morphological erosion algorithm was employed to generate coarse masks, simulating annotations that indicate regions of interest as might be marked during clinical examinations. Specifically, all images and their corresponding ground truth masks were first bilinearly interpolated to a resolution of 352 × 352 pixels and normalized to the [0, 1] range. Subsequently, these high-quality labeled masks were transformed into coarser versions through iterative morphological erosions. Each iteration applied a binary erosion operation to the designated region, progressively shrinking its boundaries. This process removes fine-grained details while preserving the overall structure, mimicking the effect of manual annotations obtained during clinical examinations. The resulting coarse mask was then downsampled to 88 × 88 pixels and provided as input to the prompt encoder, enabling the model to learn accurate segmentation at higher resolutions than those directly provided. This approach effectively generates simulated noisy annotations via multi-level erosion. It provides a robust experimental foundation for evaluating the model’s resilience to weak supervision.

Comparative experiments were conducted to evaluate the performance of various segmentation networks on the Kvasir-Seg, CVC-ClinicDB, CVC-ColonDB, CVC-300, and ETIS datasets. The evaluated methods included UNet++, TransUNet, TransFuse, CaraNet, PraNet, UACANet, Polyp_PVT, CFANet, ConDSeg, and EMCAD. The corresponding results are summarized below. Overall, the WCEDSAM model consistently outperformed all baseline methods across every evaluation metric, demonstrating superior segmentation accuracy and robustness across all datasets.

To ensure fairness and scientific rigor in the experimental comparisons, models including TransUNet, TransFuse, and Polyp_PVT were initialized with pre-trained weights, thereby fully leveraging the advantages of transfer learning. During data preprocessing, all models consistently employed standardized data augmentation strategies to mitigate any potential bias arising from differences in data handling. Concerning hyperparameter optimization, considering the variations in computational complexity and GPU memory requirements across network architectures, adaptive tuning was conducted according to available hardware resources. This approach aimed to maintain training stability while maximizing the performance potential of each model under the given hardware constraints.

#### 4.4.1. Quantitative Analysis

In benchmark experiments on the Kvasir-SEG dataset, WCEDSAM achieved a mean Dice (mDice) score of 0.9383 and a mean Intersection over Union (mIoU) of 0.9074. For comparison, Polyp-PVT—the model with the second-best performance—achieved mDice of 0.9248 and mIoU of 0.8773. Simultaneously, WCEDSAM attained precision and recall rates of 0.9484 and 0.9345, respectively, indicating superior performance in both positive sample localization accuracy and detection completeness. Other comparative methods, including PraNet and UACANet, achieved mDice scores exceeding 0.89 (0.9063 and 0.9096, respectively); however, none of their metrics matched the performance of WCEDSAM (Table 2).

On the CVC-ClinicDB dataset, the performance of all comparative models declined to varying degrees relative to Kvasir-SEG, reflecting differences in the dataset’s sample distribution. For instance, the mDice score of TransUNet decreased to 0.6729. Despite these variations in data characteristics, WCEDSAM maintained an mDice of 0.9376 and an mIoU of 0.8943, along with a precision of 0.9614 and a recall of 0.9206 (Table 3). None of the primary metrics exhibited significant fluctuations, indicating the model’s robustness across different data distributions.

In experiments conducted on the CVC-ColonDB dataset, WCEDSAM achieved an mDice score of 0.9189 and mIoU of 0.8855, with precision and recall values of 0.9304 and 0.9132, respectively. In contrast, Polyp-PVT, which ranked second, achieved an mDice score of only 0.7357. Traditional architectures, such as UNet++ and TransUNet, achieved mDice scores of 0.5153 and 0.5415, respectively, on this dataset, highlighting their limitations in handling complex background noise, whereas WCEDSAM demonstrated clear advantages in both boundary delineation and lesion region identification (Table 4).

In evaluations conducted on the CVC-300 dataset, WCEDSAM ranked first with an mDice score of 0.8961 and mIoU of 0.8284, attaining a precision of 0.9265 and a recall of 0.8704. PraNet and CFANet followed closely with mDice scores of 0.8888 and 0.8866, respectively, while Polyp-PVT achieved an mDice score of 0.8478. Overall, WCEDSAM outperformed these mainstream methods across all primary evaluation metrics.

On the ETIS dataset, widely recognized as highly challenging, WCEDSAM achieved an mDice score of 0.7765 and mIoU of 0.7326. In contrast, the top-performing comparative models, Polyp-PVT and PraNet, achieved mDice scores of 0.6485 and 0.6323, respectively. The dataset contains numerous small, low-contrast polyp samples with blurred edges. WCEDSAM’s performance on these complex samples demonstrates its capability to extract more discriminative features, effectively mitigating the diagnostic ambiguities commonly encountered in conventional models.

Based on cross-dataset evaluations across the five aforementioned datasets, WCEDSAM outperformed all comparative models across primary evaluation metrics, demonstrating high consistency and strong generalization capabilities. Notably, while achieving top-tier segmentation accuracy, WCEDSAM comprises only 15.38 million parameters and requires 9.461 gigaflops of computational cost. By comparison, ConDSeg comprises 170.25 million parameters, whereas TransUNet contains 119.38 million. This lightweight model architecture not only efficiently addresses complex medical image segmentation tasks but also substantially reduces computational overhead, rendering it highly practical for clinical applications and deployment on medical devices.

#### 4.4.2. Qualitative Analysis

**Figure 5:**
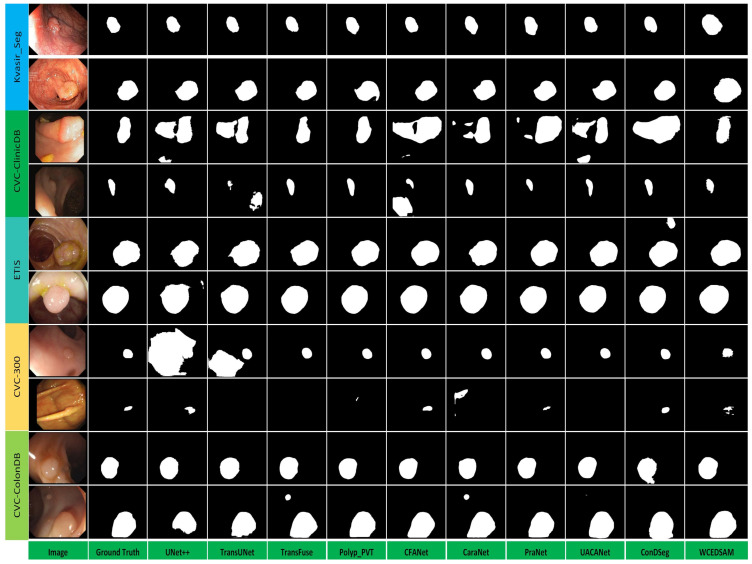
Examples of visualization outputs from the Kvasir_Seg, CVC-ClinicDB, CVC-ColonDB, CVC-300, and ETIS datasets using different deep learning models.

Figure 5 presents visualization results for cross-dataset medical image segmentation, allowing direct comparison of the segmentation performance and generalization capabilities of different models. Overall trends in the predicted masks indicate that, from the classic UNet++ to the proposed WCEDSAM model, the agreement between the segmentation results and the ground truth gradually improves. The proposed model not only restores the complete contour of the target regions more accurately but also provides finer segmentation of small polyp details; simultaneously, it effectively suppresses background noise interference while preserving the structural integrity of the polyps, thereby demonstrating superior generalization capability.

In contrast, compared with the UNet++ model, earlier architectures, such as TransUNet, exhibited clear under-segmentation or over-segmentation issues in certain test samples and demonstrated limited adaptability to the data distributions of different datasets. Other mainstream comparative models also suffer from insufficient target integrity and susceptibility to background interference, with generalization performance inferior to that of the proposed model. These visualization results confirm that, through structural optimization, module integration, and contextual modeling, the proposed model effectively enhances segmentation stability for unseen samples, making it more suitable for cross-dataset medical image segmentation tasks (Figure 5).

### 4.5. Ablation Experiment

To demonstrate the effectiveness of the proposed module, a lightweight baseline model was first established. The baseline was based on MedSAM, which was substantially reduced using deep compression techniques. Specifically, the embedding dimension of the image encoder was decreased from 768 to 384, the number of transformer layers was reduced from 12 to 6, and the number of attention heads was adjusted from 12 to 6. To preserve effective feature representation despite the model reduction, the modified image encoder was initialized with pre-trained weights from ViT-S/16. This strategy facilitates the transfer and retention of visual priors learned through large-scale pre-training, while significantly reducing the model’s parameter count. These modifications produced a parameter-efficient version of MedSAM, serving as the foundation for subsequent ablation experiments.

Accordingly, a two-part ablation study was systematically conducted. The experimental design followed an incremental approach, first evaluating the individual effects of the two proposed core modules—the WTCA module and the DSConv_ECA module—before assessing their combined impact on the segmentation performance of the model. Experiments were conducted across five benchmark datasets: Kvasir-Seg, CVC-ClinicDB, CVC-ColonDB, CVC-300, and ETIS. Segmentation performance was quantitatively assessed using metrics including the mean Dice coefficient (mDice), mean Intersection over Union (mIoU), precision, and recall, alongside the evaluation of model parameter count.

#### 4.5.1. Quantitative Analysis

Ablation experiments were conducted on the Kvasir-Seg dataset to evaluate the effectiveness of the proposed modules. The baseline model achieved an mDice of 0.9309, an mIoU of 0.9020, a precision of 0.9441, and a recall of 0.9249, with 15.22 million parameters and a computational cost of 9.370 G. The model operated at 2.65 FPS with an inference latency of 377.23 ms. Incorporation of the WTCA module improved the model’s recall to 0.9302 and precision to 0.9492, while mDice and mIoU increased to 0.9338 and 0.9041, respectively. The number of parameters remained at 15.22 million, and computational cost increased slightly to 9.384 G. The frame rate improved to 2.99 FPS, and inference time decreased to 334.13 ms. These results indicate that the WTCA module enhances the model’s ability to capture target regions, reduces false negatives, and improves inference efficiency. When the DSConv_ECA module was introduced independently, precision increased further to 0.9495, recall slightly rose to 0.9278, and mDice reached 0.9342. The number of parameters increased to 15.38 million, and computational cost rose to 9.447 G, with a frame rate of 2.92 FPS and an inference time of 342.39 ms. This demonstrates that DSConv_ECA improves the model’s accuracy in identifying lesion features by optimizing feature extraction and channel attention. When WTCA and DSConv_ECA were combined, the model achieved optimal performance, with mDice, mIoU, and recall reaching 0.9383, 0.9074, and 0.9345, respectively, while maintaining a high precision of 0.9484. The model comprised 15.38 million parameters and required 9.461 G of computational cost, operating at 3.33 FPS with an inference time of 300.50 ms. These results fully validate the synergistic effect of the two modules in enhancing segmentation overlap, reducing false negatives, and achieving an effective balance between accuracy and inference efficiency with minimal additional computational overhead (Table 5).

Ablation experiments conducted on the CVC-ClinicDB dataset further clarified the individual contributions of each module. The baseline model achieved mDice and mIoU scores of 0.9238 and 0.8695, respectively, operating at 4.73 FPS with an inference latency of 211.60 ms. Upon incorporation of the WTCA module, recall increased from 0.8945 to 0.9000, and mIoU improved to 0.8740, while precision slightly decreased to 0.9591, and FPS marginally decreased to 4.70. Inference latency remained largely unchanged at 212.62 ms. These results indicate that the WTCA module expands the model’s detection range, effectively reducing false negatives, while introducing a small number of false positives and slightly increasing computational overhead. When the DSConv_ECA module was applied independently, recall increased substantially to 0.9092, and mIoU and mDice improved to 0.8819 and 0.9312, respectively. Despite a reduction in FPS to 3.43 and an increase in inference time to 291.92 ms, these findings demonstrate that DSConv_ECA significantly enhances the representation of boundary features and suppresses background interference, even with added computational cost. Ultimately, the WCEDSAM model, integrating both modules, achieved comprehensive improvements: mDice, mIoU, and recall reached 0.9376, 0.8943, and 0.9206, respectively, while precision recovered to 0.9614. The model operated at 3.29 FPS with an inference time of 303.98 ms. These results demonstrate that the complementary effects of the two modules not only maximize lesion detection but also restore segmentation accuracy via feature calibration, achieving optimal segmentation performance within an acceptable inference time (Table 6).

Ablation experiments conducted on the CVC-ColonDB dataset further validated the effectiveness of each module. The baseline model attained mDice and mIoU scores of 0.9044 and 0.8607, respectively, with precision and recall of 0.9230 and 0.8964. It operated at 3.56 FPS with an inference latency of 280.90 ms. Following the incorporation of the WTCA module, recall improved significantly to 0.9110, with mDice and mIoU increasing to 0.9141 and 0.8766, respectively, and precision rising slightly to 0.9237. The model’s FPS increased to 4.04, and inference time decreased to 247.67 ms, indicating that WTCA effectively expands the detection range, reduces false negatives, and enhances inference efficiency. Independent application of the DSConv_ECA module increased precision to 0.9264, recall to 0.9065, and mDice and mIoU to 0.9119 and 0.8754, respectively. FPS increased to 4.29, and inference time decreased to 232.99 ms, indicating that DSConv_ECA optimizes feature extraction via depthwise separable convolutions and channel attention mechanisms, enhances lesion feature identification, and improves inference speed. When WTCA and DSConv_ECA were combined, the model achieved optimal performance, with mDice, mIoU, and recall improving to 0.9189, 0.8855, and 0.9132, respectively, and precision further increasing to 0.9304. FPS reached 4.52, and inference time decreased to 221.01 ms (Table 6). These results fully validate the synergistic effects of the two modules in enhancing segmentation overlap, reducing false negatives, and simultaneously optimizing segmentation accuracy and inference speed.

Ablation experiments conducted on the CVC-300 dataset further validated the effectiveness of the proposed modules. The baseline model achieved a recall of 0.8017, with mDice and mIoU of 0.8721 and 0.7891, respectively, operating at 2.39 FPS and an inference latency of 418.62 ms. Upon incorporation of the WTCA module alone, recall increased substantially to 0.8502, mIoU improved to 0.8198, and mDice reached 0.8906, while precision decreased slightly from 0.9650 to 0.9454. FPS increased to 3.44 and inference time decreased to 299.47 ms, demonstrating that WTCA effectively expands the detection range and identifies potential lesion areas while enhancing inference speed. These results reaffirm the primary contribution of the WTCA module in expanding detection coverage and enhancing lesion identification, although a slight reduction in precision is observed. Introduction of the DSConv_ECA module independently increased recall to 0.8365, mIoU to 0.8127, and mDice to 0.8870, while precision slightly declined to 0.9507. The model operated at 2.41 FPS with an inference time of 415.60 ms, indicating that DSConv_ECA enhances the representation of complex features through depthwise separable convolutions and channel attention, albeit with a higher computational cost. When WTCA and DSConv_ECA were combined in WCEDSAM, the model achieved optimal performance: mDice, mIoU, and recall reached 0.8961, 0.8284, and 0.8704, respectively, while precision decreased to 0.9265. The model operated at 2.04 FPS with an inference time of 490.73 ms. In medical imaging segmentation, high recall and segmentation overlap are of greater clinical importance, demonstrating that the combined modules improve sensitivity and lesion coverage, even at the expense of increased computational overhead (Table 7).

Ablation experiments conducted on the ETIS dataset further demonstrated that each module contributes substantially to performance enhancements. The baseline model achieved mDice and mIoU scores of 0.7584 and 0.7098, respectively, operating at 3.22 FPS with an inference latency of 310.60 ms. Incorporation of the WTCA module alone increased mDice to 0.7727, mIoU to 0.7279, recall from 0.7450 to 0.7583, and precision slightly to 0.8035. FPS decreased to 2.43 and inference time increased to 411.07 ms, indicating that WTCA expands detection coverage and maintains localization accuracy, albeit with additional computational cost. These results indicate that on more challenging datasets, WTCA effectively enhances the detection range while preserving precise localization, despite increased computational demands. Application of the DSConv_ECA module independently increased mDice to 0.7724, mIoU to 0.7288, recall to 0.7610, and precision to 0.8033. The model operated at 2.92 FPS with an inference time of 342.22 ms, confirming the module’s effectiveness in optimizing feature channel weights and enhancing key feature representation. These findings further corroborate the effectiveness of DSConv_ECA in enhancing feature representation and channel weighting. When the two modules were combined in the WCEDSAM model, mDice, mIoU, and recall improved to 0.7765, 0.7326, and 0.7667, respectively, with precision maintained at 0.7981. The model achieved 4.12 FPS with an inference time of 242.77 ms, demonstrating enhanced efficiency alongside accuracy. These results demonstrate that in complex, small-object scenarios, the synergy between WTCA and DSConv_ECA maximizes segmentation completeness and boundary alignment, achieving simultaneous improvements in accuracy and inference efficiency.

In summary, the ablation experiments clearly demonstrate the critical roles and complementary contributions of the WTCA and DSConv_ECA modules in polyp segmentation. The primary contribution of the WTCA module is its ability to expand the model’s receptive field over target regions via an attention mechanism, thereby significantly reducing false negatives while enhancing both recall and precision. The DSConv_ECA module enhances the model’s capacity to capture lesion boundaries and critical features through depthwise separable convolutions combined with an efficient channel attention mechanism, optimizing feature representation and further improving recall. The integrated WCEDSAM model achieved superior performance, with the highest mDice, mIoU, and recall values across four datasets of varying sizes and complexity. These results indicate that the global contextual awareness provided by WTCA and the fine-grained feature extraction of DSConv_ECA act synergistically, maximizing lesion detection completeness while maintaining precise boundary delineation. Collectively, these findings provide an efficient and robust solution for medical image segmentation tasks.

#### 4.5.2. Qualitative Analysis

**Figure 6:**
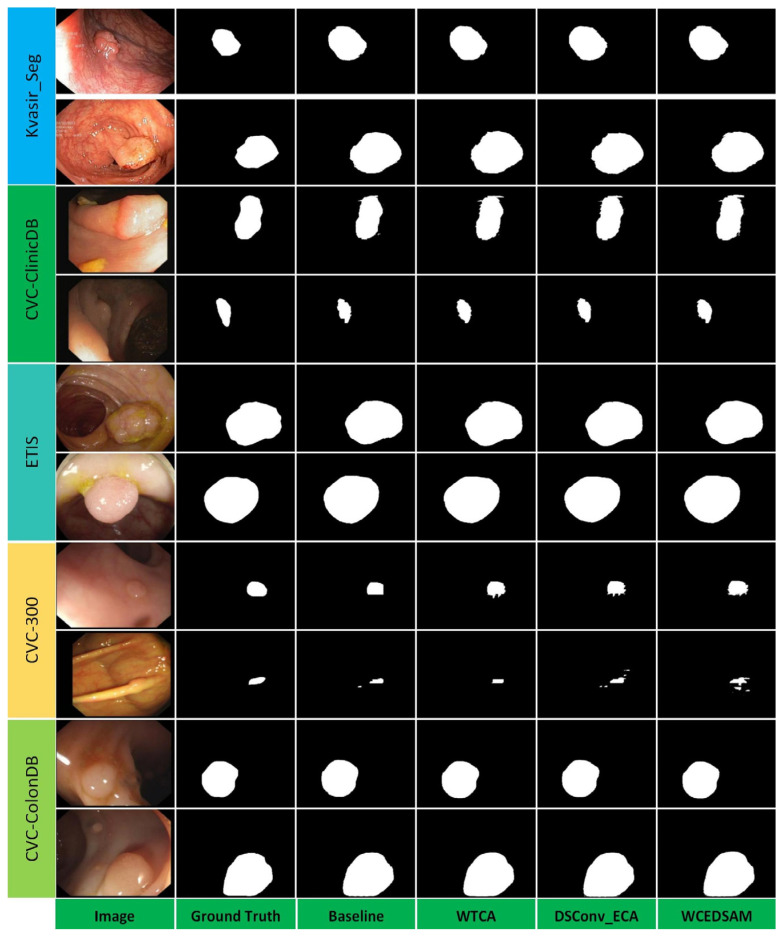
Examples of visualization outputs from ablation experiments on the Kvasir_Seg, CVC-ClinicDB, CVC-ColonDB, CVC-300, and ETIS datasets.

To objectively assess the contribution of each module to the model’s overall generalization ability, a systematic cross-domain polyp segmentation study was conducted across five publicly available datasets exhibiting substantial feature variations. The segmentation results visualized for each comparative method are presented in Figure 6. Analysis of both visual and quantitative results indicates that, although the baseline model achieves acceptable performance on intra-domain test sets, its cross-domain segmentation is suboptimal, exhibiting evident missed lesion detections and unclear segmentation boundaries. This issue is particularly pronounced in the ETIS dataset, which contains complex lesion morphologies and blurred lesion-background boundaries. Following the incorporation of the WTCA module, the model exhibited a markedly enhanced response to lesion regions, with substantial improvements in recall; however, the module occasionally led to over-segmentation in certain background areas. Subsequent inclusion of the DSConv_ECA module augmented the model’s multi-scale context information extraction, enabling more precise segmentation boundaries and enhanced robustness to image artifacts. The WCEDSAM model, integrating both modules, ultimately achieved the highest segmentation performance across five cross-domain test datasets. This approach effectively reduced false positive and false negative segmentations in target domain data while maintaining high segmentation accuracy for source domain images, with predicted lesion contours closely aligned with ground truth. Both quantitative and visual analyses demonstrate that the WTCA module possesses strong global context modeling capabilities, while the DSConv_ECA module enables adaptive calibration of multi-scale features. The combination of these modules effectively mitigates overfitting caused by domain shifts and ultimately enhances the model’s generalization capability in cross-dataset segmentation tasks (Figure 6).

## 5. Discussion

### 5.1. Experimental Results Analysis

The WCEDSAM model proposed in this study achieves an optimal balance between computational efficiency and segmentation accuracy. Quantitative analysis indicates that WCEDSAM comprises only 15.38 million parameters, substantially fewer than the 119.38 million parameters of TransUNet and the 170.25 million parameters of ConDSeg. This compact design does not compromise performance; instead, it is achieved through the effective integration of depthwise separable convolutions and efficient channel attention mechanisms. Depthwise separable convolutions substantially reduce parameter redundancy by decoupling spatial and channel-wise feature extraction, whereas efficient channel attention facilitates cross-channel information exchange with minimal computational overhead. Moreover, qualitative analysis presented in Figure 5 demonstrates that the explicit extraction and enhancement of high-frequency details, achieved through the combination of wavelet transforms and the channel-cooperative attention module, enables WCEDSAM to accurately detect blurred boundaries and fine-scale structures. Compared with the oversegmentation or undersegmentation frequently observed in models such as UNet++ and TransUNet, WCEDSAM delineates polyp contours with greater precision, further confirming the efficacy of frequency-domain decomposition in preserving edge features in medical images.

### 5.2. Main Contributions

The primary contribution of this study is the design of two core modules, WTCA and DSConv-ECA, and their integration into the lightweight MedSAM architecture. These enhancements address critical challenges in medical image segmentation, including multi-scale feature fusion, preservation of fine-grained details, and computational efficiency. In comparison with the original MedSAM, this study introduces systematic innovations in input dimensions, encoder architecture, and feature preprocessing strategies: The input resolution was reduced from 1024 × 1024 to 352 × 352, the embedding dimension of the image encoder was compressed from 768 to 384, the number of Transformer layers was decreased from 12 to 6, and the number of attention heads was reduced from 12 to 6. Despite the substantial reduction in computational overhead, the incorporation of the WTCA and DSConv-ECA modules mitigated information loss due to the lower resolution, thereby achieving an optimal balance between a lightweight design and high segmentation performance.

Existing multi-scale feature fusion methods frequently employ static operations, such as concatenation or element-wise addition, in combination with attention modules like SE [53] or CBAM [54] to modulate features along channel or spatial dimensions. However, these approaches exhibit notable limitations. The SE module compresses spatial information via global average pooling, thereby modeling only global channel dependencies while disregarding the spatial distribution of features. This limitation hinders the precise localization of small target regions. Although CBAM integrates both channel and spatial attention mechanisms, its reliance on max-pooling operations leads to the loss of substantial background information. In addition, the sequential execution of the two attention modules generates redundant computational overhead, reducing the ability to accurately differentiate foreground from background in medical images characterized by blurred lesion boundaries and low contrast. Moreover, these methods do not explicitly model frequency-domain information and fail to exploit feature differences across distinct frequency subbands, resulting in suboptimal segmentation performance for small objects and fine structural details. While the original MedSAM demonstrates strong segmentation capabilities, its high parameter count and substantial memory requirements constrain its applicability in high-resolution medical image processing. Additionally, MedSAM has not been tailored to the frequency characteristics of medical images, limiting its adaptability to multi-scale lesion segmentation.

The proposed WTCA module integrates the two-dimensional discrete wavelet transform with a channel-cooperative attention mechanism in a novel manner. The module decomposes the input features into four sub-bands—LL, LH, HL, and HH—using Haar wavelet bases, explicitly separating low-frequency contour information from high-frequency detail components. Based on the properties of each sub-band, a cross-subband channel collaborative attention mechanism was designed. Inter-channel dependencies were modeled using 1 × 1 convolutions, while residual connections and pixel rearrangement operations were incorporated to restore spatial resolution, thereby facilitating the enhancement and fusion of frequency-domain features. Compared to the original MedSAM, the incorporation of the WTCA module allows the model to effectively capture high-frequency details at reduced input resolutions, mitigating the loss of edge information due to downsampling and improving detection sensitivity for small lesions.

The DSConv-ECA module mitigates the loss of local details commonly observed during patch embedding in Transformer encoders by integrating depthwise separable convolutions with Efficient Channel Attention (ECA), thereby providing lightweight feature preprocessing prior to the encoder. In this module, depthwise convolutions are employed to independently extract spatial features from each channel, followed by 1 × 1 point convolutions to fuse cross-channel information. The parameter count of this design is approximately one-ninth that of standard convolutions. Subsequently, an ECA module is incorporated, utilizing adaptive one-dimensional convolutions to capture local cross-channel interactions. This design avoids the information loss typically associated with dimension reduction in the fully connected layers of the SE module. Compared with the direct patch embedding employed in the original MedSAM, the DSConv-ECA module enables effective local feature extraction and channel-adaptive weighting prior to entry into the Transformer encoder. This significantly enhances the encoder’s capacity to represent edges and fine-grained structures, while maintaining a minimal parameter overhead.

In summary, the WTCA and DSConv-ECA modules constitute a complementary framework, integrating global modeling in the frequency domain with local enhancement in the spatial domain. The WTCA module facilitates cross-scale frequency-domain feature fusion through wavelet decomposition and channel-cooperative attention, whereas the DSConv-ECA module supports efficient local feature extraction and channel-adaptive weighting using depthwise separable convolutions and ECA. The synergy of these two modules, coupled with a lightweight redesign of the core MedSAM architecture—including an input size of 352 × 352, an embedding dimension of 384, and six Transformer layers—allows WCEDSAM to reduce its parameter count by approximately 95% relative to the original MedSAM, resulting in a total of only 15.38 million parameters. Simultaneously, WCEDSAM attains state-of-the-art segmentation performance across five public datasets, including Kvasir-SEG and CVC-ClinicDB, substantially outperforming mainstream models such as TransUNet (119.38 M), ConDSeg (170.25 M), and other comparable architectures. This study introduces a novel paradigm for lightweight design in medical image segmentation, particularly suitable for computationally constrained clinical environments. The proposed approach achieves an optimal balance between segmentation accuracy and computational efficiency, overcoming the limitations of the original MedSAM, which was challenging to deploy on standard clinical GPUs (e.g., 8 GB VRAM) due to its substantial computational requirements.

### 5.3. Limitations and Future Work

Although WCEDSAM has demonstrated outstanding performance on public datasets, and the semi-automated interaction mechanism could theoretically reduce the annotation burden for clinicians, significant challenges remain in translating these results into clinical practice. In the present study, qualitative evaluation relied predominantly on visual comparisons with ground truth labels and did not undergo rigorous validation by clinical endoscopy experts. In real-world clinical settings, the accuracy of segmentation results is reflected not only in pixel-level overlap metrics, such as the Dice coefficient, but also in their ability to support clinical decision-making—for instance, whether segmentation boundaries influence the evaluation of polyp invasion depth. The absence of expert validation diminishes confidence in the model’s applicability in clinical practice. Future studies should incorporate multi-center, multi-physician evaluations of image interpretation and employ metrics such as Kappa coefficients and time-saving rates, calculated using computational models, to more objectively assess the clinical utility of these approaches.

Although WCEDSAM exhibits strong generalization capabilities, a critical evaluation of its potential risks and biases remains essential. Cross-domain testing on datasets such as ETIS indicates that the model may still generate missed detections or boundary inaccuracies when polyps present extremely flat morphologies, such as laterally spreading tumors, exhibit minimal color contrast relative to the surrounding mucosa, or appear in endoscopic images with pronounced specular reflections and motion blur. Under these conditions, exclusive reliance on frequency-domain features is inadequate for the accurate differentiation of lesions from artifacts.

Although the model demonstrated strong performance in cross-domain tests, including CVC-ColonDB and ETIS, the image feature distributions in the training datasets Kvasir-SEG and CVC-ClinicDB may contain inherent biases, such as relatively ideal lighting conditions and standard polyp morphologies. Such biases may result in suboptimal model performance when applied to images from previously unseen endoscopic devices or specific patient populations. Moreover, public datasets are generally curated and lack the extensive representation of normal frames and complex backgrounds characteristic of real-world clinical settings, which deviates from the long-tail distribution observed in clinical practice.

The present study primarily focuses on comparisons among task-specific models, including UNet++ and PraNet. With the rapid development of foundational models in medical image segmentation, general-purpose large models, such as SAM2 and its medical adaptations, have exhibited substantial potential for zero-shot and few-shot segmentation. Although WCEDSAM offers advantages in model efficiency, the absence of comparisons with the most recent large-scale foundational models under highly complex scenarios limits current understanding of its standing relative to the state of the art.

This study presents several limitations that should be addressed in future research. WCEDSAM utilizes a semi-automatic segmentation paradigm, and its performance is partially dependent on the quality of the input coarse prompt mask. In the present experiment, the prompt mask was generated by morphologically eroding the ground truth labels, representing an idealized simulation. In contrast, in real-world clinical practice, coarse outlines manually drawn by physicians may be highly arbitrary and incomplete. It remains to be determined whether the model can robustly compensate for substantial deviations in the initial input from the target region while still generating accurate segmentation results.

To address common practical constraints, such as limited GPU memory, the standard input image size was set to 352 × 352 pixels. Comparative experiments were conducted to assess the model’s sensitivity to varying input resolutions. The results demonstrated that an input resolution of 352 × 352 yields superior segmentation accuracy relative to smaller resolutions, including 256 × 256 and 224 × 224. This input size directly affects the trade-off between computational complexity and segmentation accuracy. WCEDSAM employs an architecture that integrates depth-separable convolutions with a windowed attention mechanism, rendering the model sensitive to feature map dimensions. Excessively large input sizes (e.g., 512 × 512) substantially increase the number of key-value pairs in self-attention computations, resulting in exponential growth in memory usage and inference time, thereby undermining the objective of a lightweight implementation. Conversely, excessively small inputs (e.g., 256 × 256) lead to the loss of fine details, such as lesion or organ boundaries, a limitation that is particularly pronounced in high-resolution medical images. The 352 × 352 input size represents a compromise between the 1024 × 1024 resolution of the original MedSAM and ultra-lightweight resolutions, preserving essential anatomical features while keeping peak memory usage within acceptable limits for standard clinical GPUs (e.g., 8 GB VRAM) and maintaining per-image inference latency below the threshold for real-time interaction. At this resolution, the computational load remains relatively low, while the information content is adequate to support effective model training. Consequently, this choice constitutes an experimentally validated design decision, balancing the model’s lightweight characteristics with its performance. Nonetheless, high resolution is critical for diagnosing early-stage micro-polyps, particularly those smaller than 5 mm. Downsampling inevitably causes the loss of fine-scale texture and edge information, thereby limiting the model’s ability to detect ultrafine lesions. Mechanisms for enhancing or adapting resolution during multi-scale testing should be explored to mitigate the information loss associated with fixed resolution.

The current model was evaluated using static images, whereas an actual colonoscopy involves a dynamic video-streaming process. In clinical workflows, models are required not only to deliver high-precision single-frame segmentation but also to satisfy real-time requirements, including a stable frame rate and temporal consistency, to prevent severe flickering of segmentation masks in the video. Extending WCEDSAM from static image processing to end-to-end real-time video segmentation and integrating it into the graphics workstation of an endoscopic system constitutes a critical step toward clinical implementation.

## 6. Conclusions

This study proposes a novel WCEDSAM model, built upon a condensed MedSAM backbone, which addresses key challenges in lesion segmentation in medical images, including multi-scale variations, blurred edges, and the omission of small objects. These challenges are mitigated through the integration of wavelet transformations, channel-cooperative attention, depthwise separable convolutions, and ECA modules. Comprehensive experiments on the Kvasir_Seg dataset demonstrate that WCEDSAM exhibits high segmentation accuracy, robust performance, and broad generalizability, significantly outperforming several mainstream segmentation models, including UNet++, Polyp_PVT, PraNet, and CFANet. The model employs a semi-automatic approach, wherein physicians provide an initial rough outline as a prompt. Following this input, the model performs automatic fine-grained segmentation based on the provided preliminary guidance. This approach substantially reduces the manual effort previously required to annotate every individual point in interactive segmentation processes. Furthermore, it enables efficient and effective batch-wise processing of medical images, minimizing physicians’ cognitive load. Overall, the model provides significant practical value in clinical settings, offering a partially automated and efficient solution for large-scale medical image analysis and patient evaluation.

The model WCEDSAM, as a semi-automatic segmentation framework, demonstrates broad applicability in medical image analysis. Firstly, the successful validation of the model highlights the effectiveness of combining wavelet transforms with attention mechanisms to enhance feature representation. This approach may be extended to other segmentation tasks across different medical imaging modalities beyond those evaluated in the current study. Secondly, since most existing models have primarily targeted static images, it is important to investigate the applicability of these methods to dynamic medical image sequences. Such an extension could capture temporal variations in disease progression and improve longitudinal analysis. Moreover, although the model performs effectively with limited data, its generalizability could be further enhanced through semi-supervised training strategies. This approach enables the model to leverage unlabeled medical data efficiently, thereby reducing dependence on extensive labeled datasets. In summary, the integration of high-performance, semi-automated segmentation systems into clinical diagnostic workflows could facilitate a synergistic “physician–AI” partnership. Such integration is expected to enhance healthcare efficiency and support precise, individualized treatment strategies.

## Figures and Tables

**Table 1:** Basic Information of five polyp datasets.

Dataset	Resolution	Total Samples	Usage
Kvasir_SEG	332 × 487~1920 × 1072	1000	split 8:2
CVC-ClinicDB	384 × 288	612	split 8:2
ETIS	1225 × 966	196	Test Only
CVC-ColonDB	574 × 500	380	Test Only
CVC-300	574 × 500	60	Test Only

**Table 2:** Comparison of performance results for various models on the Kvasir-Seg and CVC-ClinicDB datasets. The bolded entries in the table represent the highest performance metrics.

Model	Kavsir_Seg						CVC-ClinicDB						Parameter	Flops
	mDice	mIoU	Precision	Recall	FPS	Interval Time	mDice	mIoU	Precision	Recall	FPS	Interval Time		
UNet++	0.8706	0.8011	0.9024	0.8783	31.64	31.61 ms	0.7004	0.6078	0.7192	0.7801	**32.50**	30.77 ms	47.19 M	**153.194 G**
TransUNet	0.8586	0.7895	0.8940	0.8695	22.35	44.74 ms	0.6729	0.5813	0.6563	0.7690	24.20	41.33 ms	119.38 M	14.408 G
TransFuse	0.8921	0.8320	0.9143	0.9010	20.53	48.72 ms	0.7387	0.6618	0.7628	0.7701	20.61	48.51 ms	57.68 M	17.525 G
CaraNet	0.8735	0.8059	0.9164	0.8744	14.03	71.27 ms	0.7090	0.6178	0.7154	0.7950	14.65	68.25 ms	16.37 M	11.184 G
PraNet	0.9063	0.8544	0.9378	0.9013	15.50	64.50 ms	0.8091	0.7261	0.8002	0.8756	17.54	57.00 ms	72.01 M	69.232 G
UACANet	0.9096	0.8618	0.9348	0.9072	11.47	87.18 ms	0.8039	0.7142	0.8486	0.8212	11.05	90.49 ms	69.16 M	59.524 G
Polyp_PVT	0.9248	0.8773	0.9378	0.9309	17.75	56.33 ms	0.8537	0.7760	0.8779	0.8699	18.38	54.42 ms	25.11 M	10.005 G
CFANet	0.9101	0.8559	0.9455	0.9025	23.54	42.48 ms	0.7879	0.6968	0.7898	0.8719	23.21	43.09 ms	27.76 M	18.702 G
ConDSeg	0.8945	0.8394	0.9136	0.9069	24.97	40.04 ms	0.7731	0.6822	0.7565	0.8816	23.51	42.54 ms	**170.25 M**	82.675 G
EMCAD	0.8255	0.7568	0.8919	0.8326	10.23	97.75 ms	0.6583	0.5670	0.7422	0.7101	10.98	91.09 ms	21.57 M	10.905 G
WCEDSAM	**0.9383**	**0.9074**	**0.9484**	**0.9345**	**3.33**	**300.50 ms**	**0.9376**	**0.8943**	**0.9614**	**0.9206**	3.29	**303.98 ms**	15.38 M	9.461 G

**Table 3:** Comparison of performance results for various models on the CVC-ColonDB test datasets.

Model	CVC-ColonDB						Parameter	Flops
	mDice	mIoU	Precision	Recall	FPS	Interval Time		
UNet++	0.5153	0.4437	0.5795	0.5304	30.56	32.72 ms	47.19 M	**153.194 G**
TransUNet	0.5415	0.4760	0.5862	0.5576	23.07	43.35 ms	119.38 M	14.408 G
TransFuse	0.6405	0.5633	0.6755	0.6848	25.38	39.40 ms	57.68 M	17.525 G
CaraNet	0.5778	0.5087	0.6008	0.5968	20.37	49.09 ms	16.37 M	11.184 G
PraNet	0.6429	0.5734	0.6804	0.6711	16.76	59.68 ms	72.01 M	69.232 G
UACANet	0.6547	0.5829	0.7156	0.6623	11.98	83.50 ms	69.16 M	59.524 G
Polyp_PVT	0.7357	0.6498	0.7774	0.7879	17.69	56.54 ms	25.11 M	10.005 G
CFANet	0.5794	0.5239	0.6296	0.5697	34.91	28.65 ms	27.76 M	18.702 G
ConDSeg	0.5480	0.4693	0.5994	0.5710	25.45	39.29 ms	**170.25 M**	82.675 G
EMCAD	0.5438	0.4743	0.6297	0.5406	10.70	93.49 ms	21.57 M	10.905 G
WCEDSAM	**0.9189**	**0.8855**	**0.9304**	**0.9132**	**4.52**	**221.01 ms**	15.38 M	9.461 G

**Table 4:** Comparison of performance results for various models on the CVC-300 and ETIS test datasets.

Model	CVC-300						ETIS						Parameter	Flops
	mDice	mIoU	Precision	Recall	FPS	Interval Time	mDice	mIoU	Precision	Recall	FPS	Interval Time		
UNet++	0.2086	0.1274	0.1485	0.8571	**30.56**	32.72 ms	0.4264	0.3658	0.4858	0.4543	27.27	36.67 ms	47.19 M	**153.194 G**
TransUNet	0.3122	0.2265	0.2346	0.8808	24.44	40.92 ms	0.3823	0.3261	0.3974	0.4547	24.03	41.61 ms	**119.38 M**	14.408 G
TransFuse	0.7288	0.6371	0.6741	0.8326	20.79	48.10 ms	0.4803	0.4156	0.4749	0.5604	20.13	49.68 ms	57.68 M	17.525 G
CaraNet	0.4750	0.3923	0.4905	0.6475	19.03	52.55 ms	0.3842	0.3397	0.3851	0.4241	14.74	67.86 ms	16.37 M	11.184 G
PraNet	0.8888	0.8095	0.8802	0.9167	16.44	60.84 ms	0.6323	0.5717	0.6523	0.6521	17.40	57.48 ms	72.01 M	69.232 G
UACANet	0.8708	0.8116	0.8739	0.8804	11.36	88.03 ms	0.5776	0.5141	0.6443	0.5972	11.30	88.52 ms	69.16 M	59.524 G
Polyp_PVT	0.8478	0.7753	0.8762	0.8536	19.18	52.14 ms	0.6485	0.5786	0.6401	0.7535	18.04	55.43 ms	25.11 M	10.005 G
CFANet	0.8866	0.8149	0.8606	0.9442	28.26	35.38 ms	0.5233	0.4784	0.5474	0.5320	31.38	31.87 ms	27.76 M	18.702 G
ConDSeg	0.8008	0.7030	0.7707	0.8849	23.66	42.27 ms	0.4672	0.4113	0.4828	0.5036	25.21	39.67 ms	170.25 M	82.675 G
EMCAD	0.7547	0.6583	0.7928	0.7808	10.67	93.70 ms	0.3576	0.2969	0.5179	0.3175	10.71	93.37 ms	21.57 M	10.905 G
WCEDSAM	**0.8961**	**0.8284**	**0.9265**	**0.8704**	2.04	**490.73 ms**	**0.7765**	**0.7326**	**0.7981**	**0.7667**	**4.12**	**242.77 ms**	15.38 M	9.461 G

**Table 5:** Comparison of performance results from ablation experiments on the Kvasir-Seg and CVC-ClinicDB datasets.

Model	Kavsir_Seg						CVC-ClinicDB						Parameter	Flops
	mDice	mIoU	Precision	Recall	FPS	Interval Time	mDice	mIoU	Precision	Recall	FPS	Interval Time		
baseline	0.9309	0.9020	0.9441	0.9249	2.65	**377.23 ms**	0.9238	0.8695	0.9603	0.8945	**4.73**	211.60 ms	15.22 M	9.370 G
WTCA	0.9338	0.9041	0.9492	0.9302	2.99	334.13 ms	0.9261	0.8740	0.9591	0.9000	4.70	212.62 ms	15.22 M	9.384 G
DSConv_ECA	0.9342	0.9035	**0.9495**	0.9278	2.92	342.39 ms	0.9312	0.8819	0.9586	0.9092	3.43	291.92 ms	15.38 M	9.447 G
WCEDSAM	**0.9383**	**0.9074**	0.9484	**0.9345**	**3.33**	300.50 ms	**0.9376**	**0.8943**	**0.9614**	**0.9206**	3.29	**303.98 ms**	**15.38 M**	**9.461 G**

**Table 6:** Comparison of performance results from ablation experiments on the CVC-ColonDB datasets.

Model	CVC-ColonDB						Parameter	Flops
	mDice	mIoU	Precision	Recall	FPS	Interval Time		
baseline	0.9044	0.8607	0.9230	0.8964	3.56	**280.90 ms**	15.22 M	9.370 G
WTCA	0.9141	0.8766	0.9237	0.9110	4.04	247.67 ms	15.22 M	9.384 G
DSConv_ECA	0.9119	0.8754	0.9264	0.9065	4.29	232.99 ms	15.38 M	9.447 G
WCEDSAM	**0.9189**	**0.8855**	**0.9304**	**0.9132**	**4.52**	221.01 ms	**15.38 M**	**9.461 G**

**Table 7:** Comparison of performance results from ablation experiments on the CVC-300 and ETIS datasets.

Model	CVC-300						ETIS						Parameter	Flops
	mDice	mIoU	Precision	Recall	FPS	Interval Time	mDice	mIoU	Precision	Recall	FPS	Interval Time		
baseline	0.8721	0.7891	**0.9650**	0.8017	2.39	418.62 ms	0.7584	0.7098	0.8004	0.7450	3.22	310.60 ms	15.22 M	9.370 G
WTCA	0.8906	0.8198	0.9454	0.8502	**3.44**	299.47 ms	0.7727	0.7279	**0.8035**	0.7583	2.43	**411.07 ms**	15.22 M	9.384 G
DSConv_ECA	0.8870	0.8127	0.9507	0.8365	2.41	415.60 ms	0.7724	0.7288	0.8033	0.7610	2.92	342.22 ms	15.38 M	9.447 G
WCEDSAM	**0.8961**	**0.8284**	0.9265	**0.8704**	2.04	**490.73 ms**	**0.7765**	**0.7326**	0.7981	**0.7667**	**4.12**	242.77 ms	**15.38 M**	**9.461 G**

## Data Availability

Our research data supporting the findings of this study are derived from publicly archived datasets. Specifically, the datasets used include Kvasir_Seg (available at https://datasets.simula.no/kvasir-seg/, accessed on 20 January 2026), CVC-ClinicDB (available at https://www.kaggle.com/datasets/orvile/cvc-clinicdb, accessed on 20 January 2026), CVC-ColonDB (available at https://www.kaggle.com/datasets/longvil/cvc-colondb, accessed on 20 January 2026), CVC-300 (available at https://www.kaggle.com/datasets/nourabentaher/cvc-300, accessed on 20 January 2026), and ETIS (available at https://www.kaggle.com/datasets/nguyenvoquocduong/etis-laribpolypdb, accessed on 20 January 2026). All these datasets are openly accessible, and no new data were generated or restricted due to privacy or ethical concerns in this study.

## Abbreviations

The following abbreviations are used in this manuscript:

CNN	Convolutional Neural Network
LSTM	Long Short-Term Memory
IWT	Integer Wavelet Transform
MLP	Multilayer Perceptron
WTCA	Wavelet Transform and Channel Collaborative Attention
ViT	Vision Transformer
ECA	Efficient Channel Attention
